# Association of infertility and recurrent pregnancy loss with the risk of dementia

**DOI:** 10.1007/s10654-024-01135-3

**Published:** 2024-06-18

**Authors:** Chen Liang, Annette J. Dobson, Hsin-Fang Chung, Yvonne T. van der Schouw, Sven Sandin, Elisabete Weiderpass, Gita D. Mishra

**Affiliations:** 1 School of Public Health, University of Queensland, Public Health Building, 288 Herston Road, Herston, Brisbane, QLD 4006 Australia; 2 Julius Center for Health Sciences and Primary Care, University Medical Center Utrecht, University Utrecht, Utrecht, The Netherlands; 3 Department of Medical Epidemiology and Biostatistics, Karolinska Institutet, Stockholm, Sweden; 4 International Agency for Research on Cancer, World Health Organization, Lyon, France

**Keywords:** Infertility, Miscarriage, Stillbirth, Dementia, Cohort study

## Abstract

Emerging evidence has shown the association between female reproductive histories (e.g., menarche age, parity, premature and early menopause) and the risk of dementia. However, little attention has been given to infertility and pregnancy loss. To examine the associations of infertility, recurrent miscarriages, and stillbirth with the risk of dementia, this study used data from four cohorts in the International Collaboration for a Life Course Approach to Reproductive Health and Chronic Disease Events. Women with data on at least one of the reproductive exposures of interest, dementia, and all covariates were included. Histories of infertility, miscarriage, and stillbirth were self-reported. Dementia (including Alzheimer’s disease) was identified through surveys, aged care, pharmaceutical, hospital, and death registry data. Cause-specific Cox regression models were used to estimate the hazard ratios of dementia, accounting for well-established risk factors of dementia, study variability, and within-study correlation. Overall, 291,055 women were included at a median (interquartile range) age of 55.0 (47.0–62.0) at baseline. During the median (interquartile range) follow-up period of 13.0 (12.0–14.0) years, 3334 (1.2%) women developed dementia. Compared to women without stillbirth, a history of recurrent stillbirths (≥ 2) was associated with 64% higher risk of dementia (adjusted hazard ratio = 1.64, 95% confidence interval: 1.46–1.85). Compared to women without miscarriage, women with recurrent miscarriages (≥ 3) were at 22% higher risk of dementia (adjusted hazard ratio = 1.22, 95% confidence interval: 1.19–1.25). These findings suggest that recurrent stillbirths is a risk factor for dementia and may need to be considered in risk assessment of dementia in women.

**Supplementary Information:**

The online version contains supplementary material available at 10.1007/s10654-024-01135-3.

## Introduction

Globally, dementia is the fifth leading cause of death in women [1]. In 2019, dementia was estimated to have caused over one million deaths and 17.7 million disability-adjusted life years (DALYs) among women, which were substantially higher than the figures for men (0.6 million deaths and 10.6 million DALYs) [2, 3]. The death rates and DALY of dementia in women aged 50–59 years, 60–69 years, and ≥ 70 years were also higher than those in men, indicating that the female predominance may not be simply due to the longer lifespan [3]. Some risk factors for dementia are well established, such as lower education, smoking, diabetes mellitus, hypertension, depression, and pre-existing stroke, but these factors are insufficient to explain the excess burden of dementia in women [4]. Sex-specific risk factors need to be investigated.

A growing number of studies suggest female reproductive histories, such as later age at menarche, nulliparity, premature and early menopause (Supplementary Table 1), as the risk factors of dementia [5–13]. These studies suggest shorter cumulative exposure to oestrogen as a key underlying mechanism. However, little attention has been given to infertility and pregnancy loss (i.e., miscarriage and stillbirth), which are often accompanied by hormonal changes (e.g., low estrogen). Additionally, systematic diseases, such as diabetes and hypertension, have been identified as risk factors for infertility and pregnancy loss, and for the development of dementia [14–16]. Therefore, it is plausible to hypothesize that women with these fertility issues may have a higher risk of dementia. As far as we know, there were three previous studies exploring the association of infertility, miscarriage, or stillbirth with dementia (Supplementary Table 1) [5, 6, 11]. These studies did not reveal an association with miscarriage or infertility, and the results on stillbirth were inconsistent. However, study limitations, such as inappropriate exclusion criteria (e.g., excluding women without children), inadequate follow-up (e.g., up to a median age of 49 years), and insufficient adjustment (e.g., lacking adjustment for chronic conditions during follow-up period), compromised their findings.

Therefore, in this study, individual-level data pooled from four cohorts contributing to the International Collaboration for a Life Course Approach to Reproductive Health and Chronic Disease Events (InterLACE) were used [17]. The aim was to assess the association of infertility, miscarriage, and stillbirth with the risk of dementia adjusting for well-established risk factors of dementia.

## Methods

### Data source and study population

The InterLACE consortium is composed of 27 observational studies, pooling individual-level data of over 850,000 women from 12 countries. The design of the InterLACE has been published previously [18]. Among these observational studies, four cohorts from four countries (Australia, Netherlands, UK, and Sweden) with data on at least one of the fertility issues (i.e., infertility, miscarriage, or stillbirth) and the outcome (dementia, including Alzheimer’s disease) were included: the Australian Longitudinal Study on Women’s Health 1946-51 cohort (ALSWH-mid), the Dutch Prospect-EPIC Utrecht in the European Prospective Investigation into Cancer and Nutrition (Prospect-EPIC), UK Biobank, and the Swedish Women’s Lifestyle and Health Study (WLH). Study characteristics are presented in Table 1. Women with data on fertility issues, dementia, and covariates were included (Supplementary Fig. 1).

**Table 1:** Characteristics of the four studies in the InterLACE consortium

Study	Country	Number	Baseline year	Age, median (interquartile range)	
				Baseline	Last follow-up
ALSWH-mid	Australia	11,770	1996	47.6 (46.3, 48.9)	70.0 (69.0, 72.0)
Prospect-EPIC	Netherlands	15,015	1993–1997	56.2 (51.8, 61.6)	72.7 (68.2, 78.0)
UK Biobank	UK	221,765	2006–2010	58.0 (51.0, 63.0)	71.0 (63.0, 76.0)
WLH	Sweden	42,505	1991–1992	40.0 (35.0, 45.0)	58.0 (53.0, 63.0)

ALSWH-mid: the Australian Longitudinal Study on Women’s Health 1946-51 cohort; Prospect-EPIC: the Utrecht contribution to the European Prospective Investigation into Cancer and Nutrition cohort, the Netherlands; WLH: the Swedish Women’s Lifestyle and Health Study

### Exposures

The histories of infertility, miscarriage, and stillbirth were retrospectively collected through questionnaires at baseline or follow-up surveys (Supplementary Table 2). Women were identified as infertile if they reported an experience of not being able to conceive after one year (or longer) of unprotected sex, medical consultation for infertility, or diagnosis or treatment of infertility by a physician. The outcome of each pregnancy (i.e., live birth, miscarriage, or stillbirth), the number of miscarriages, and the number of stillbirths were also collected. The number of miscarriages was grouped into four categories (0, 1, 2, and ≥ 3), and the number of stillbirths was grouped into three categories (0, 1, and ≥ 2) [19, 20]. Recurrent miscarriages was defined as three or more miscarriages, and recurrent stillbirths was defined as two or more stillbirths, which could be interspersed with live births.

### Outcome

Information on dementia was obtained through self-reported data or health administrative data, including aged care assessment, pharmaceutical scripts, hospital admissions, and death registrations (Supplementary Table 2). All the studies provided hospital admission data, and three of them (ALSWH-mid, Prospect-EPIC, and UK Biobank) also had death registry data. Hospital admission and death registry data were coded using the 9th or 10th versions of the International Classification of Diseases (ICD-9 and ICD-10). ICD-9 codes 290.0, 290.1, 290.2, 290.4, 290.8, 290.9, 331.0, 331.1 and ICD-10 codes F00, F01, F03, G30, G31.0 were used to define dementia, including Alzheimer’s disease [21]. In addition to ICD codes, ALSWH-mid and Prospect-EPIC identified dementia cases through questionnaire items on physician-diagnosed dementia and dementia treatment, and UK Biobank identified dementia cases through centre visit assessment. ALSWH-mid also identified the cases from aged care and pharmaceutical data (medication for Alzheimer’s disease).

### Covariates

Covariates were collected at cohort entry, except for the incidences of hypertension, diabetes mellitus, and stroke (Supplementary Table 2). The incidences of hypertension, diabetes mellitus, and stroke were collected until the end of follow-up. For Asian women, body mass index (BMI) was categorized as underweight (< 18.5 kg/m^2^), normal (18.5–22.9 kg/m^2^), overweight (23–27.4 kg/m^2^), and obese (≥ 27.5 kg/m^2^). For other women, the categories of BMI were defined as underweight (< 18.5 kg/m^2^), normal (18.5–24.9 kg/m^2^), overweight (25.0–29.9 kg/m^2^) and obese (≥ 30.0 kg/m^2^). Other covariates were race/ethnicity (Caucasian, Asian, and other), education level (≤ 10, 11–12, and > 12 years), age at last birth (≤ 20, 21–25, 26–30, 31–35, > 35 years old), smoking status (current smoker, and former or non-smoker), alcohol intake (none, monthly, weekly, and daily), depression (yes and no), hypertension (yes and no), diabetes mellitus (yes and no), and stroke (yes and no). For the main analysis, the categories of Asian and other races were combined due to the small number of women.

### Statistical analysis

Baseline characteristics were presented as numbers and percentages for categorical variables, and as medians and interquartile ranges (IQRs) for continuous variables. Kaplan Meier plots were created to compare the times to dementia between exposed and unexposed women, and the log-rank test was conducted to assess the difference between these times. Cause-specific Cox regression models were used to examine the association between infertility, miscarriage, stillbirth, and risk of dementia with age as the timescale. Dementia was the event of interest, and women without dementia were censored at death or the end of follow-up. Hazard ratios (HRs) and 95% confidence intervals (CIs) were estimated. Difference between studies was taken into account by including study as a fixed categorical covariate. Robust variance estimators were used to account for potential within-study correlation. All models were adjusted for fixed covariates (i.e., race/ethnicity, education level, smoking status, and BMI at baseline) and time-dependent covariates (i.e., incidence of hypertension, diabetes, and stroke up to the end of follow-up) [4, 15, 16, 22, 23]. Analyses for miscarriage and stillbirth were restricted to women who had ever been pregnant.

To examine the robustness of findings, multiple sensitivity analyses were conducted. First, Fine and Gray competing risk models for the sub-distribution hazard of dementia were fitted. Deaths from other causes were taken as competing events. That was because the prevalence of dementia increases sharply with age especially after the age of 70, and participants might die from other causes before the occurrence of dementia, which precluded the occurrence of dementia [1, 24]. Second, analyses were restricted to women who ever had children, to exclude the influence of nulliparity. Third, analyses were restricted to women without a history of gestational diabetes or gestational hypertension, to exclude the influence of these two pregnancy complications. Fourth, cause-specific models were additionally adjusted for age at last birth, alcohol intake, and depression status, which were not included in the main analysis due to high proportion of missing data. The associations of infertility, miscarriage, and stillbirth with dementia were also examined in each cohort separately. All statistical analyses were performed using SAS version 9.4 (SAS Institute Inc, Cary, NC). All tests of hypotheses were conducted at the two-sided 5% level of significance. No adjustments were made for multiple testing.

## Results

Overall, 291,055 women at a median (IQR) age of 55.0 (47.0, 62.0) years from four cohorts were included and were followed up for a median (IQR) of 13.0 (12.0–14.0) years. The percentages of women who experienced infertility, miscarriage, and stillbirth were 17.9%, 25.4%, and 3.2%, respectively. The baseline characteristics of women with and without these reproductive issues were compared in Table 2. By the end of follow-up, 3334 (1.2%) women had developed dementia at a median (IQR) age of 75.0 (71.0–78.0). Among these women with dementia, 2511 were identified through a single data source (2019 from hospital admission data, 299 from death registry data, 82 from pharmaceutical data and 111 from other data sources), while 758 and 65 cases were identified through two and three or more data sources, respectively. The characteristics of women with and without dementia were provided in supplementary Table 3. Due to missing data, 16.6%, 4.9%, and 4.0% women were excluded in analyses of infertility, miscarriage, and stillbirth, respectively (Supplementary Fig. 1). Excluded women were more likely to be less-educated, current smokers, and have diabetes or stroke (Supplementary Table 4).

**Table 2:** Characteristics of study participants

Characteristics	Infertility (*N* = 67,010)		Miscarriage (*N* = 244,608)		Stillbirth (*N* = 234,947)	
	Never	Ever	Never	Ever	Never	Ever
Exposure, No. (%)						
	55,028 (82.1%)	11,982 (17.9%)	182,434 (74.6%)	62,174 (25.4%)	227,359 (96.8%)	7,588 (3.2%)
Race/ethnicity, No. (%)						
Caucasian	54,729 (99.5%)	11,936 (99.6%)	174,024 (95.4%)	58,972 (94.9%)	216,967 (95.4%)	6,787 (89.4%)
Others	299 (0.5%)	46 (0.4%)	8,410 (4.6%)	3,202 (5.2%)	10,392 (4.6%)	801 (10.6%)
Education level, No. (%)						
≤ 10	20,025 (36.4%)	3,826 (31.9%)	97,045 (53.2%)	30,088 (48.4%)	117,728 (51.8%)	4,519 (59.6%)
11–12	15,812 (28.7%)	3,499 (29.2%)	25,034 (13.7%)	8,978 (14.4%)	31,500 (13.9%)	903 (11.9%)
> 12	19,191 (34.9%)	4,657 (38.9%)	60,355 (33.1%)	23,108 (37.2%)	78,131 (34.4%)	2,166 (28.6%)
Smoking status, No. (%)						
Never or past smoker	43,901 (79.8%)	9,145 (76.3%)	164,606 (90.2%)	55,350 (89.0%)	205,403 (90.3%)	6,656 (87.7%)
Current smoker	11,127 (20.2%)	2,837 (23.7%)	17,828 (9.8%)	6,824 (11.0%)	21,956 (9.7%)	932 (12.3%)
Body-mass index, No. (%)						
Underweight	788 (1.4%)	202 (1.7%)	1,262 (0.7%)	463 (0.7%)	1,504 (0.7%)	47 (0.6%)
Normal	33,646 (61.1%)	7,733 (64.5%)	69,842 (38.3%)	23,743 (38.2%)	86,479 (38.0%)	2,338 (30.8%)
Overweight	15,111 (27.5%)	2,982 (24.9%)	68,541 (37.6%)	22,834 (36.7%)	85,572 (37.6%)	2,951 (38.9%)
Obese	5,483 (10.0%)	1,065 (8.9%)	42,789 (23.5%)	15,134 (24.3%)	53,804 (23.7%)	2,252 (29.7%)
Hypertension, No. (%)						
No	36,555 (66.4%)	8,550 (71.4%)	114,913 (63.0%)	38,969 (62.7%)	144,879 (63.7%)	4,073 (53.7%)
Yes	18,473 (33.6%)	3,432 (28.6%)	67,521 (37.0%)	23,205 (37.3%)	82,480 (36.3%)	3,515 (46.3%)
Diabetes mellitus, No. (%)						
No	49,719 (90.4%)	10,795 (90.1%)	168,540 (92.4%)	56,638 (91.1%)	210,819 (92.7%)	6,659 (87.8%)
Yes	5,309 (9.7%)	1,187 (9.9%)	13,894 (7.6%)	5,536 (8.9%)	16,540 (7.3%)	929 (12.2%)
Stroke, No. (%)						
No	52,971 (96.3%)	11,557 (96.5%)	176,453 (96.7%)	59,895 (96.3%)	220,059 (96.8%)	7,178 (94.6%)
Yes	2,057 (3.7%)	425 (3.6%)	5,981 (3.3%)	2,279 (3.7%)	7,300 (3.2%)	410 (5.4%)

Women from three studies (i.e., ALSWH-mid, WLH, and Prospect-EPIC), three studies (i.e., ALSWH-mid, UK Biobank, and Prospect-EPIC), and two studies (i.e., UK Biobank and Prospect-EPIC) were included in the analysis for infertility, miscarriage, and stillbirth, respectively. Body-mass index (kg/m^2^): For Asian, the classifications of body mass index are < 18.5 (underweight), 18.5–22.9 (normal), 23-27.4 (overweight), and ≥ 27.5 (obese). For others, the classifications of body mass index are < 18.5 (underweight), 18.5–24.9 (normal), 25-29.9 (overweight), and ≥ 30 (obese). Information on exposure, race/ethnicity, education level, smoking status, and body-mass index was collected at baseline, when information on hypertension, diabetes, and stroke was updated to the end of follow-up

In the analysis of infertility, 67,010 women were included, and 501 developed dementia. There was insufficient evidence to establish an association between infertility and dementia (adjusted HR = 1.09, 95%CI: 0.81–1.46, Table 3, Supplementary Fig. 2).

**Table 3:** Association between Infertility, miscarriage, stillbirth, and dementia

Exposure		Person-year	Sample size	Event	IR	Crude model HR (95%CI)	Adjusted model HR (95%CI)
History of infertility	Never	3,504,824	55,028	431	1.23	Ref.	Ref.
	Ever	737,923	11,982	70	0.95	1.08 (0.79,1.49)	1.09 (0.81,1.46)
History of miscarriage	Never	12,738,716	182,434	2,423	1.90	Ref.	Ref.
	Ever	4,291,693	62,174	764	1.78	0.98 (0.95,1.02)	0.97 (0.95,1.00)
Number of miscarriages	0	12,738,716	182,434	2,423	1.90	Ref.	Ref.
	1	3,065,128	44,317	524	1.71	0.94 (0.91,0.97)	0.94 (0.91,0.96)
	2	786,986	11,434	139	1.77	0.97 (0.85,1.12)	0.96 (0.83,1.11)
	≥ 3	435,644	6,369	97	2.23	1.30 (1.23,1.37)	1.22 (1.19,1.25)
History of stillbirth	Never	15,815,662	227,359	2,851	1.80	Ref.	Ref.
	Ever	537,885	7,588	145	2.70	1.21 (1.12,1.31)	1.09 (1.03,1.15)
Number of stillbirths	0	15,815,662	227,359	2,851	1.80	Ref.	Ref.
	1	467,222	6,580	117	2.50	1.12 (1.05,1.20)	1.01 (0.97,1.05)
	≥ 2	69,911	998	28	4.01	1.91 (1.79,2.03)	1.64 (1.46,1.85)

IR: incidence rate (per 10 000 person-years). HR: hazard ratio. CI: confidence interval. Women (*N* = 67,010) from ALSWH-mid, WLH, and Prospect-EPIC were included in the analysis for infertility, and women with and without infertility developed dementia at a median (IQR) age of 66.0 (59.0, 74.0) and 72.0 (67.0, 78.0), respectively. Women (*N* = 244,608) from ALSWH-mid, UK Biobank, and Prospect-EPIC were included in the analysis for miscarriage, and women with 0, 1, 2, and ≥ 3 miscarriages had dementia at a median (IQR) age of 75.0 (72.0, 78.0), 75.0 (71.0, 78.0), 75.0 (70.0, 78.0), and 75.0 (69.0, 78.0), respectively. Women (*N* = 234,947) from UK Biobank and Prospect-EPIC were included in the analysis for stillbirth, and women with 0, 1 and ≥ 2 stillbirths developed dementia at a median (IQR) age of 76.0 (72.0, 79.0), 76.0 (73.0, 78.0), and 75.5 (70.5, 78.0), respectively. Models were adjusted for race, education level, smoking status, body-mass index, hypertension, diabetes, and stroke, when hypertension, diabetes, and stroke were included as time-varying covariates. Study variability and within-study correlation were taken into account by including study as a covariate and using robust variance estimators in all models

Among women who had ever been pregnant, 244,608 were included in the analysis of miscarriage, and 3187 developed dementia. Compared to women without miscarriage, women with recurrent miscarriages (≥ 3) had a modestly higher risk of dementia (adjusted HR = 1.22, 95%CI: 1.19–1.25; Table 3, Supplementary Figs. 3–4).

A total of 234,947 women who had ever been pregnant were included in the analysis of stillbirth, and 2996 developed dementia. Compared to women without stillbirth, women who experienced a stillbirth were at higher risk of dementia in the crude model (HR = 1.21, 95% CI: 1.12–1.31), which was attenuated in the adjusted model (HR = 1.09, 95%CI: 1.03–1.15, Table 3, Supplementary Fig. 5). However, analysis of the number of stillbirths revealed a stronger association for recurrent stillbirths (≥ 2) (adjusted HR = 1.64, 95%CI 1.46–1.85; Table 3, Supplementary Fig. 6).

### Sensitivity analysis

First, Fine and Gray competing risk models were fitted, and the results were similar to the main analysis (Supplementary Table 5). Second, analysis restricted to women who had children did not alter the results (Supplementary Table 6). Third, analysis restricted to women without gestational hypertension or gestational diabetes revealed similar results to the main analysis (Supplementary Table 7). Fourth, when models with additional adjustment of age at last birth, alcohol intake, and depression status were fitted, the estimated HRs of infertility, miscarriage, and stillbirth decreased slightly, and the 95% CIs became wider due to smaller sample size (Supplementary Table 8). The associations of infertility, miscarriage, and stillbirth with dementia in each cohort were provided separately (supplementary Figs. 7–9).

## Discussion

This pooled analysis of 291,055 women from four prospective cohorts showed a 1.64-fold increased risk of dementia among women with a history of recurrent stillbirths (≥ 2). The association for recurrent miscarriages (≥ 3) was weaker, with around 22% increased risk. However, there was insufficient evidence to support an association between infertility and dementia risk.

To our knowledge, there has been only one previous cohort study reporting an association between infertility and dementia. In the study by Andolf et al., 1,128,709 Swedish women with at least one live birth were enrolled, and a lower risk of dementia was observed among women with secondary infertility [5]. Women with infertility were identified through ICD codes (N970, N978, N979) only, which failed to include women who experienced infertility but did not seek medical help. In the present study, women without children were included, and a history of infertility was defined through infertility diagnosis, fertility treatment, or failing to conceive after one year (or longer) of unprotected sex, which could have included primary infertility and infertility due to male factors. Compared to Andolf et al.’s study, a much higher proportion of women were considered infertile in the present study (17.9% vs. 2.4%). Nevertheless, the number of women included was insufficient to provide evidence that all-cause infertility was associated with dementia.

This study suggested a modest association between recurrent miscarriages (≥ 3) and dementia. Three previous studies examined the association between miscarriage and dementia. Andolf et al., Basit et al., and Gong et al. recruited 1,128,709, 1,243,957, and 273,240 women from Sweden, Denmark, and the UK, respectively, and found no evidence on the association between miscarriage (single or recurrent) and dementia [5, 6, 11]. In the study by Andolf et al., ICD-10 codes of O262 (pregnancy care for patient with recurrent pregnancy loss) and N96 (recurrent pregnancy loss) were used to identify recurrent miscarriages although these codes taken together were not specific for recurrent miscarriages [5]. In the studies by Basit et al. and Gong et al., the number of miscarriages was classified as 0, 1, and ≥ 2, while in the present study the number of miscarriages was classified as 0, 1, 2, and ≥ 3 [6, 11]. There is no consensus on the definition of recurrent miscarriages (≥ 2 or ≥ 3) [22]. The present study showed that ≥ 3 miscarriages were associated with dementia risk, as had also been observed in previous studies on stroke and premature all-cause death [25, 26].

The present study also revealed an elevated risk of dementia among women with recurrent stillbirths. Only two previous studies have investigated the association between stillbirth and dementia, and their findings were inconsistent. Basit et al. showed women with a history of stillbirth were at higher risk of dementia (HR = 1.86, 95%CI: 1.28–2.71), but Gong et al. reported no association with single or recurrent stillbirths [6, 11]. Both studies had limitations. Women were followed up to a median age of 49 in Basit et al.’s study [6], which is insufficient for the occurrence of dementia [1]. Besides, control for confounding may have been inadequate. Basit et al. did not control for smoking, Gong et al. failed to adjust for education level, and neither of them took into account the incidence of chronic conditions (i.e., hypertension, diabetes, and stroke) during the follow-up period [6, 11]. In the present study, women were followed up to a median (IQR) age of 69.0 (62.0–75.0) years, and models were adjusted for more potential confounders, which provided a more precise estimate for the association between recurrent stillbirths and dementia.

The effect size for recurrent stillbirths on the risk of dementia [1.64 (1.46, 1.85)] was comparable to the effect sizes of obesity, hypertension, and diabetes [1.6 (1.3, 1.9), 1.6 (1.2, 2.2) and 1.5 (1.3, 1.8)] in the 2020 Lancet report [4]. The magnitude of the effect size supports the consideration of recurrent stillbirths as a risk factor for dementia in women. Health professionals should be aware of the potentially increased risk of dementia among women with a history of recurrent stillbirths, and tailor preventive measures accordingly.

The observed associations of recurrent miscarriages and recurrent stillbirths with dementia may be explained by endothelial dysfunction. Previous studies on the elevated risk of cardiovascular disease and stroke among women with recurrent pregnancy loss supported this hypothesis: endothelial dysfunction could not only cause placentation-related defects, leading to pregnancy loss, but also persist after a complicated pregnancy, which may result in adverse health conditions for the development of dementia in later life [27–31]. Cerebrovascular endothelial cell dysfunction can activate glia and the inflammatory environment in the brain, resulting in cerebral blood flow dysregulation and blood-brain barrier damage [27]. In this process, synapses, neuronal axons, and white matter are compromised, leading to cognitive impairment [27]. Meanwhile, oestrogen and progesterone are fundamental regulators of mitochondrial function and neuroplasticity in the female central nervous system [32, 33]. Women with pregnancy loss may have inadequate level of oestrogen or progesterone. Mitochondrial dysfunction and declining neuroplasticity, owing to oestrogen and progesterone deficiency, may provide a link between pregnancy loss and dementia. Loss of the neuroprotective effects of female sex hormones (e.g., protecting the blood-brain barrier, down-regulating inflammatory cascade, and limiting cellular necrosis and apoptosis) would also elevate the risk of dementia [34, 35]. Additionally, stroke, diabetes, and depression, which are more prevalent among women with pregnancy loss, may promote the development of dementia through cerebrovascular lesions or structural changes, increased glucocorticoid steroid levels, inflammatory changes (e.g., increases in IL-6 and TNF), toxic effects of hyperglycaemia (e.g., oxidative stress imbalance) and hyperinsulinemia (e.g., interfering with Aβ metabolism) [31, 36–39]. Other factors, such as homocysteine levels and abnormal maternal immune responses, may be involved as well [40–44]. Miscarriages or stillbirths due to these factors are likely to recur if these factors persist during the reproductive stage. However, miscarriages or stillbirths caused by chromosomal abnormalities or infections are not necessarily recurring and are unlikely to be linked to the development of dementia. This may partly explain the null associations of single miscarriage and single stillbirth with dementia. These hypotheses are mainly from observational studies, and molecular studies are needed in the future to confirm the underlying mechanisms linking pregnancy loss and dementia.

### Limitations

This study had several limitations. First, information about the histories of infertility, miscarriage, and stillbirth was collected through questionnaires, which might introduce recall bias. Previous studies have shown that self-reported infertility and pregnancy loss had sensitivities of 73.5% and 72.0%, respectively [45, 46]. Therefore, recall bias might have limited influence on the results. Second, the present study could not separate female and male infertility. It has been reported that 20–30% of infertility cases are due to male factors only, and the rest are due to female factors only or both male and female factors, indicating the majority due to female factors [15]. Third, there might be unidentified dementia cases or delayed detection of dementia cases. Diagnosis of dementia is often long and difficult, and outpatient data which might have identified the condition earlier was not included in the present study. It is possible that some dementia cases were not detected or detected long after the dementia onset. Fourth, the associations for dementia subtypes (i.e., Alzheimer’s dementia and other dementia) were not explored due to data limitations. The distribution of dementia subtypes (Alzheimer’s dementia, other dementia, or mixed types) differed considerably between studies, possibly due to the inconsistent use of criteria or age differences of the women. Furthermore, dementia subtype may become more complex with increasing age; for example, of the 3334 dementia cases, 2801 were identified through hospital admission data, and the admission diagnoses of dementia subtypes changed among 549 women during the follow-up period. Fifth, the covariates were all collected at midlife. We were unable to adjust for important reproductive age risk factors for infertility and pregnancy loss, including smoking, alcohol intake, and BMI. Uncontrolled confounders might mask the association for dementia. Sixth, some women were excluded due to missing data, which could lead to sample bias. Besides, women in the present study were mainly Caucasian, which limited the generalisability of current findings to other populations. Seventh, the present study did not collect data on hormonal or blood biomarkers, and future studies with such data might shed more light on the underlying mechanisms. Finally, the study involved many statistical tests, and we did not adjust for the multiplicity. Still, our main hypothesis involved five independent tests. Using a Bonferroni approach, *p*-values for miscarriage and stillbirth (as ordinal categorical variables) were less than 0.05/5.

## Conclusions

In summary, this study indicated that the risk of dementia was increased among women with a history of recurrent stillbirths. The association with recurrent miscarriages was weaker, and there was insufficient power to assess any association with all-cause infertility. This finding suggests that recurrent stillbirths should be considered an early marker for later risk of dementia. More cohort studies in other populations are needed to confirm this finding, and molecular studies are required to reveal the exact biological mechanisms.

### Electronic supplementary material

Below is the link to the electronic supplementary material.

Supplementary Material 1

## Data Availability

The InterLACE data are not freely available. The anonymised dataset is governed by a Collaborative Research Agreement among several institutions. Those interested in collaborating on the project can contact the scientific committee at interlace@uq.edu.au.
