# Supplementary material for: Association of infertility and recurrent pregnancy loss with the risk of dementia
Source: Eur J Epidemiol. 2024 Jun 18;39(7):785–93. doi: 10.1007/s10654-024-01135-3 (PMC11343804; doi:10.1007/s10654-024-01135-3)
Supplement: Supplementary file 1 — Supplementary Material 1 [file 10654_2024_1135_MOESM1_ESM.docx]

**Association of infertility and recurrent pregnancy loss with the risk of dementia**

Chen LIANG Ms.^1^ (0000-0002-0023-1066), Annette J. DOBSON PhD^1^ (0000-0003-4956-0124), Hsin-Fang CHUNG PhD^1^ (0000-0003-3261-5942), Yvonne T. VAN DER SCHOUW PhD^2^ (0000-0002-4605-435X), Sven SANDIN PhD^3^ (0000-0001-6994-4884), Elisabete WEIDERPASS PhD^4^ (0000-0003-2237-0128), Gita D. MISHRA PhD^1^ (0000-0001-9610-5904)

1. University of Queensland, School of Public Health, Queensland, Australia

2. Julius Center for Health Sciences and Primary Care, University Medical Center Utrecht, University Utrecht, Utrecht, The Netherlands

3. Department of Medical Epidemiology and Biostatistics, Karolinska Institutet, Stockholm, Sweden

4. International Agency for Research on Cancer, World Health Organization, Lyon, France

**Correspondence to:** Annette J. Dobson.

Address: School of Public Health, The University of Queensland, Public Health Building, 288 Herston Road, Herston, Brisbane, Queensland 4006, Australia.

Phone number: +61 7 336 55393

Email: [a.dobson@sph.uq.edu.au](mailto:a.dobson@sph.uq.edu.au)

**Contents**

[Previous studies 3](#_Toc153802384)

[Table 1. Summary of previous studies on female reproductive histories and dementia 3](#_Toc153802385)

[Characteristics 7](#_Toc153802386)

[Figure 1. Flow chart of sample for analysis of the association between infertility, miscarriage, stillbirth, and dementia in the InterLACE consortium 7](#_Toc153802387)

[Table 2. Ascertainment of exposures and outcomes 8](#_Toc153802388)

[Table 3. Characteristics of women with and without dementia 10](#_Toc153802389)

[Table 4. Characteristics of women included and excluded from the analyses 11](#_Toc153802390)

[Kaplan-Meier survival plot with log-rank test 12](#_Toc153802391)

[Figure 2. Kaplan-Meier survival curves comparing the rate of dementia according to the history of infertility 12](#_Toc153802392)

[Figure 3. Kaplan-Meier survival curves comparing the rate of dementia according to the history of miscarriage 12](#_Toc153802393)

[Figure 4. Kaplan-Meier survival curves comparing the rate of dementia according to the number of miscarriages 13](#_Toc153802394)

[Figure 5. Kaplan-Meier survival curves comparing the rate of dementia according to the history of stillbirth 13](#_Toc153802395)

[Figure 6. Kaplan-Meier survival curves comparing the rate of dementia according to the number of stillbirths 14](#_Toc153802396)

[Sensitivity analyses 15](#_Toc153802397)

[Table 5. Sensitivity analysis for the association between infertility, miscarriage, stillbirth, and dementia estimated by Fine and Gray competing risk model 15](#_Toc153802398)

[Table 6. Sensitivity analysis for the association between infertility and dementia restricting to women with children 15](#_Toc153802399)

[Table 7. Sensitivity analysis for the association between infertility and dementia restricting to women without gestational hypertension or gestational diabetes 16](#_Toc153802400)

[Table 8. Sensitivity analysis for the association between miscarriage and dementia with additional adjustment of age at last birth, alcohol intake and depression status 17](#_Toc153802401)

[Association in single study 18](#_Toc153802402)

[Figure 7. Association between infertility and dementia in each study 18](#_Toc153802403)

[Figure 8. Association between miscarriage and dementia in each study 18](#_Toc153802404)

[Figure 9. Association between stillbirth and dementia in each study 19](#_Toc153802405)

# **Previous studies**

## **Table 1. Summary of previous studies on female reproductive histories and dementia**

| **Author, year** | **Study population** | **Sample size** | **Menarche age** | **Fertility issue** | **Parity** | **Menopause age** | **Proposed mechanisms** |
| --- | --- | --- | --- | --- | --- | --- | --- |
| **Dementia** | | | | | | | |
| Hao W, 2023^1^ | Women aged 40-69 years old in UK from 2006-2010 | 160,080 |  |  |  | Ref.=46-50  ≤40: 1.36 (1.01, 1.83) 41-45: 1.19 (1.03, 1.37) | - Estrogen exposure; - Adverse cardiovascular disease risk factors. |
| Fu CY, 2023^2^ | Women in UK Biobank | 253,611 |  |  | Ref.=2  ≥5: 1.41 (1.01, 1.96) |  | - Estrogen exposure; - Coronary heart disease and stroke; - Social support and pressure; |
| DiBiase RM, 2023^3^ | Women born between 1921 and 1945 living in four US communities | 7921 |  |  | Ref=2  0-1: 0.85 (0.71, 1.02)  ≥5: 0.86 (0.73, 1.01) |  | - Social support. |
| Gemmil A, 2022^4^ | US women over the age of 50 | 7942 |  |  | Ref.=1  ≥4: 1.13 (0.95, 1.35) |  | - |
| Gong J, 2022^5^ | Women aged 40-69 years old in UK from 2006-2010 | 273,240 | Ref.=13  <12: 1.20 (1.08, 1.34)  ≥15: 1.19 (1.07, 1.34) | Miscarriage  Ref.=0  1: 0.90 (0.78, 1.03)  ≥2: 1.02 (0.83, 1.25)  Stillbirth  Ref.=0  1: 1.15 (0.88, 1.49)  ≥2: 1.27 (0.66, 2.45) | Ref.=2  0: 1.18 (1.04, 1.33)  ≥4: 1.14 (0.98, 1.33) | Ref.=50  <47: 1.32 (1.15, 1.51) | - Estrogen exposure; - Socioeconomic status; - Psychological distress. |
| Andolf E, 2020^6^ | All women giving birth in Sweden between 1973 and 1993. | 1,128,709 |  | Infertility  Ref.=never  ever: 0.80 (0.68, 0.96)  Recurrent miscarriages:  Ref.=never  ever: 0.94 (0.54,1.61) |  |  | - |
| Yoo JE, 2020^7^ | Korean post-menopausal women without dementia were enrolled from 2009 to 2014 | 4,696,633 | Ref.=13-14  ≤12: 1.07 (1.01, 1.14)  ≥17: 1.15 (1.13, 1.16) |  | Ref.=0  ≥2: 1.01 (0.99, 1.08) | Ref.<40  40-44: 0.96 (0.93, 0.98) | - Estrogen exposure. |
| Bae JB, 2020^8^ | Community-dwelling women aged 60 or older from 6 members of the Cohort Studies of Memory in an International Consortium | 9756 |  |  | Ref.=1-4  0: 0.84 (0.63, 1.12)  ≥5: 1.30 (1.02, 1.67) |  | - Estrogen exposure; - Higher average glucose level; - Low HDL. |
| Bae JB, 2020^9^ | Community-dwelling women aged 60 or older from 11 members of the Cohort Studies of Memory in an International Consortium | 14,792 |  |  | Ref.=1  0: 0.92 (0.67, 1.27)  ≥5: 1.47 (1.10, 1.94) |  | - Estrogen exposure; - Coronary heart disease, stroke, diabetes mellitus, and depression; - Socioeconomic disadvantages. |
| Basit S, 2019^10^ | Women with pregnancy in Denmark in the period 1997-2015 | 1,243,957 |  | Miscarriage  Ref.=0  1: 0.99 (0.87, 1.12)  ≥2: 1.06 (0.84, 1.35)  Stillbirth  Ref.=never  ever: 1.86 (1.28, 2.71) |  |  | - Endothelial dysfunction and vascular pathology; - Abnormal maternal immune response; - Pregnancy induced estrogen change (↑) - APOE4 variants; - Hypertension, obesity, diabetes, and depression; - High homocysteine level. |
| Gilsanz P, 2019^11^ | Female members aged 40-55 of Kaiser Permanente Northern California between 1964 and 1973 | 4074 | Ref.=13  ≤10: 0.89 (0.63, 1.26)  16-17: 1.23 (1.01, 1.50) |  |  | Ref.=51-55  31-40: 1.20 (0.98, 1.45) 41-45: 1.29 (1.12, 1.49) | - Estrogen and progesterone exposure. |
| Gilsanz P, 2019^11^ | Female members aged 50-55 of Kaiser Permanente Northern California between 1964 and 1973 | 3191 |  |  |  | Ref.=51-55  31-40: 1.26 (0.99, 1.60)  41-45: 1.42 (1.21, 1.67) | - Estrogen and progesterone exposure. |
| Prince MJ, 2017^12^ | Women aged 65 years and over in urvan sites in catchment areas | 9428 |  |  | Nulliparity  Ref.=never  ever: 1.16 (0.86, 1.56) | Premature menopause  Ref.=never  ever: 1.19 (0.91, 1.55) | - |
| Geerlings MI, 2001^13^ | Women aged 55 years or older without dementia at baseline (1990-1993) | 3601 | Ref. ≥15  ≤12: 1.18 (0.82, 1.70)  14: 0.89 (0.57, 1.29) |  |  |  | - Estrogen exposure (APOE 4 allele interaction with estrogen). |
| **Vascular Dementia** | | | | | | | |
| Hao W, 2023^1^ | Women aged 40-69 years old in UK from 2006-2010 | 160,080 |  |  |  | Ref.=46-50  ≤40: 1.59 (0.88, 2.88) 41-45: 1.25 (0.92, 1.70) | - Estrogen exposure; - Adverse cardiovascular disease risk factors. |
| Fu CY, 2023^2^ | Women in UK Biobank | 253,611 |  |  | Ref.=2  ≥5: 2.26 (1.38, 3.69) |  |  |
| Andolf E, 2020^6^ | All women giving birth in Sweden between 1973 and 1993. | 1,128,709 |  | Infertility  ref.=never  ever: 1.32 (0.81, 2.14)  Recurrent miscarriages:  Ref.=never  ever: 0.64 (0.09,4.59) |  |  | - |
| Yoo JE, 2020^7^ | Korean post-menopausal women without dementia were enrolled from 2009 to 2014 | 4,696,633 | Ref.=13-14  ≤12: 1.14 (0.96, 1.34)  ≥17: 1.16 (1.10, 1.22) |  | Ref.=0  ≥2: 1.09 (0.96, 1.24) | Ref.<40  40-44: 0.91 (0.83, 0.99) | - Estrogen exposure. |
| Basit S, 2019^10^ | Women with ≥ pregnancy in Denmark in the period 1997-2015 | 1,243,957 |  | Miscarriage  Ref.=0  1: 1.14 (0.71, 1.85)  2: 1.93 (0.88, 4.24)  ≥3: 3.56 (1.12, 11.3)  Stillbirth  Ref.=never  ever: 2.29 (0.56, 9.31) |  |  | - Endothelial dysfunction and vascular pathology; - Abnormal maternal immune response; - Pregnancy induced estrogen change (↑) - APOE4 variants; - Hypertension, obesity, diabetes, and depression; - High homocysteine level. |
| **Unspecified Dementia** | | | | | | | |
| Basit S, 2019^10^ | Women with ≥ pregnancy in Denmark in the period 1997-2015 | 1,243,957 |  | Miscarriage  Ref.=0  1: 0.99 (0.83, 1.17)  2: 0.82 (0.55, 1.21)  ≥3: 1.01 (0.50, 2.04)  Stillbirth  Ref.=never  ever: 2.06 (1.27, 3.32) |  |  | - Endothelial dysfunction and vascular pathology; - Abnormal maternal immune response; - Pregnancy induced estrogen change (↑) - APOE4 variants; - Hypertension, obesity, diabetes, and depression; - High homocysteine level. |
| **Alzheimer’s Dementia** | | | | | | | |
| Hao W, 2023^1^ | Women aged 40-69 years old in UK from 2006-2010 | 160,080 |  |  |  | Ref.=46-50  ≤40: 1.48 (0.98, 2.25)  41-45: 1.04 (0.84, 1.29) | - Estrogen exposure;   Adverse cardiovascular disease risk factors. |
| Fu CY, 2023^2^ | Women in UK Biobank | 253,611 |  |  | Ref.=2  ≥5: 1.45 (1.13, 1.87) |  | - Estrogen exposure; - Coronary heart disease and stroke;   Social support and pressure; |
| Andolf E, 2020^6^ | All women giving birth in Sweden between 1973 and 1993. | 1,128,709 |  | Infertility  ref.=never  ever: 0.90 (0.66,1.23)  Recurrent miscarriages:  Ref.=never  ever: 1.12 (0.46,2.69) |  |  | - |
| Yoo JE, 2020^7^ | Korean post-menopausal women without dementia were enrolled from 2009 to 2014 | 4,696,633 | Ref.=13-14  ≤12: 1.06 (0.99, 1.14) ≥17: 1.14 (1.12, 1.16) |  | Ref.=0  ≥2: 1.05 (1.00, 1.10) | Ref.<40  40-44: 0.96 (0.93, 0.99) | - Estrogen exposure. |
| Bae JB, 2020^8^ | Community-dwelling women aged 60 or older from 6 members of the Cohort Studies of Memory in an International Consortium | 9756 |  |  | Ref.=1-4  0: 0.87 (0.61, 1.24)  ≥5: 1.20 (0.90, 1.61) |  | - Estrogen exposure; - Higher average glucose level;   Low HDL. |
| Jang H, 2019^14^ | Women ≥60 years of age | 3549 |  |  | Ref.=1-4  0: 1.78 (0.31, 10.31) ≥5: 1.72 (1.05, 2.81) |  | - Pregnancy induced estrogen change (low level or repeated extreme upregulation; withdraw after delivery) and decreased postmenopausal serum free oestradiol with multiple childbirths; - Hypertension, coronary heart disease, and diabetes mellitus; - Socioeconomic status. |
| Basit S, 2019^10^ | Women with ≥ pregnancy in Denmark in the period 1997-2015 | 1,243,957 |  | Miscarriage  Ref.=0  1: 0.96 (0.78, 1.18)  2: 1.27 (0.85, 1.89)  ≥3: 0.82 (0.31, 2.21)  Stillbirth  Ref.=never  ever: 1.55 (0.80, 2.99) |  |  | - Endothelial dysfunction and vascular pathology; - Abnormal maternal immune response; - Pregnancy induced estrogen change (↑) - APOE4 variants; - Hypertension, obesity, diabetes, and depression; - High homocysteine level. |
| Geerlings MI, 2001^13^ | Women aged 55 years or older without dementia at baseline (1990-1993) | 3601 | Ref.≥15  ≤12: 1.15 (0.77, 1.72) 14: 0.76 (0.48, 1.21) |  |  |  | - Estrogen exposure (APOE 4 allele interaction with estrogen). |

# **Characteristics**

## **Figure 1. Flow chart of sample for analysis of the association between infertility, miscarriage, stillbirth, and dementia in the InterLACE consortium**


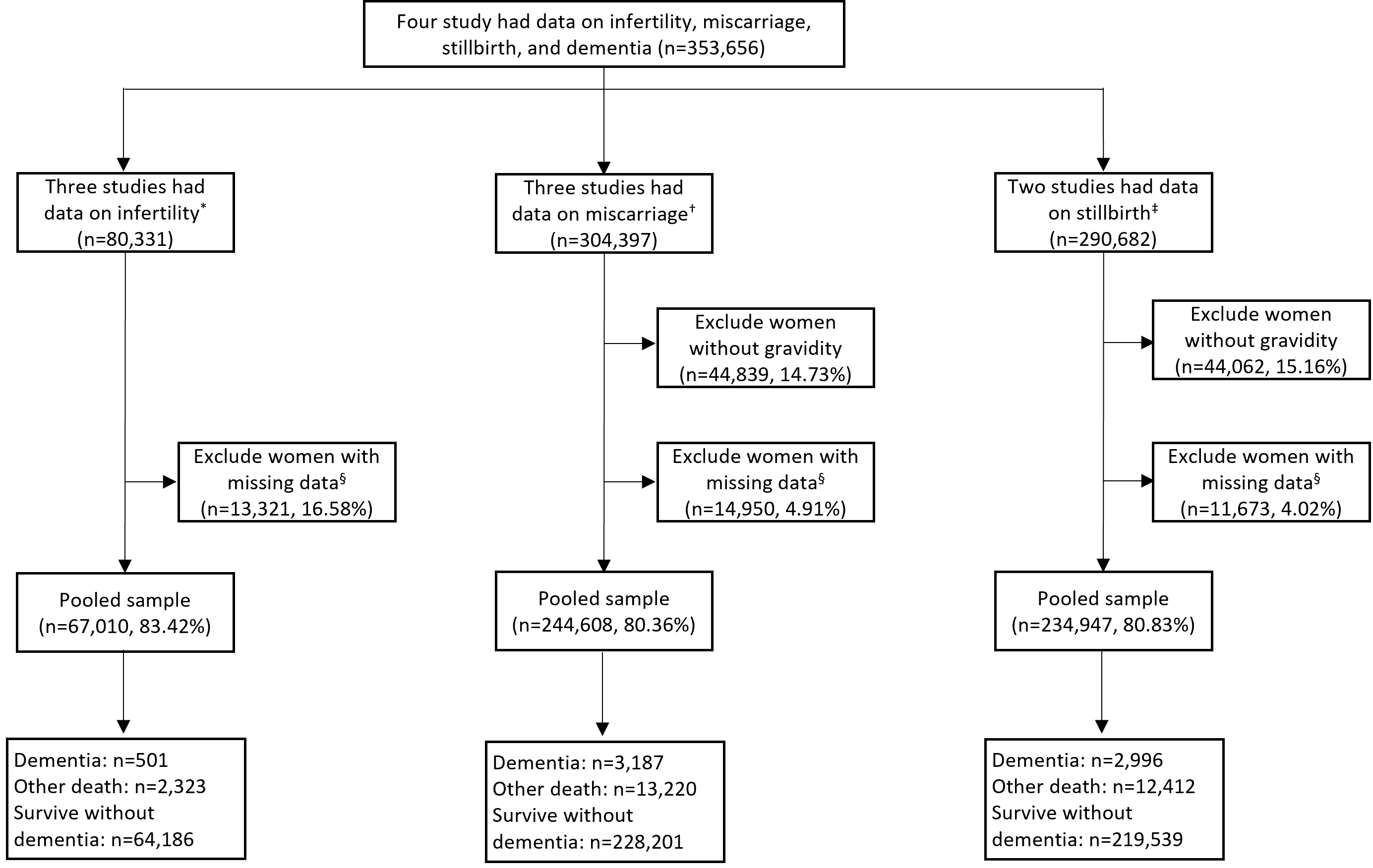


The four included studies were Australian Longitudinal Study on Women’s Health 1946-51 cohort (ALSWH-mid), the Dutch Prospect-EPIC Utrecht in the European Prospective Investigation into Cancer and Nutrition (Prospect-EPIC), UK Biobank, and the Swedish Women’s Lifestyle and Health Study (WLH). ^*^ ALSWH-mid, Prospect-EPIC, and WLH. ^†^ ALSWH-mid, Prospect-EPIC, and UK Biobank. ^‡^ Prospect-EPIC and UK Biobank. ^§^ Women with missing data on exposures (infertility, miscarriage, or stillbirth), outcomes (dementia), survival time, or covariate factors (race/ethnicity, education level, smoking status, body-mass index, hypertension, diabetes, and stroke) were excluded. For miscarriage and stillbirth, women who had never been pregnant were additionally excluded.

## **Table 2. Ascertainment of exposures and outcomes**

| Study | Infertility | Miscarriage | Stillbirth | Dementia | Covariates |
| --- | --- | --- | --- | --- | --- |
| ALSWH-mid  (Survey 1-9) | Questionnaire (survey 3):  1.unsuccessfully to get pregnant? (For 12 months or more)  2.diagnosed as infertile  3.treatment for infertility | Questionnaire (survey 1): number of miscarriages | Not available | Questionnaire (survey 7-9): diagnosis or treatment of dementia  Hospital admission data (up to December 2019): ICD 9 (290.0, 290.1, 290.2, 290.4, 290.8, 290.9, 331.0, 331.1) and ICD 10 (F00, F01, F03, G30, G31.0)  Aged care data (up to December 2019): 50 (dementia in Alzheimer’s disease), 51 (vascular dementia), and 532 (unspecified dementia)  Pharmaceutical Benefits Scheme data (up to June 2020): N06DA01 (Tacrine), N06DA02 (Donepezil), N06DA03 (Rivastigmine), N06DA04 (Galantamine), N06DA52 (Donepezil and memantine), N06DA53 (Donepezil, memantine and Ginkgo folium), and N06DX01 (Memantine)  Death registry data (up to December 2019): ICD 9 (290.0, 290.1, 290.2, 290.4, 290.8, 290.9, 331.0, 331.1) and ICD 10 (F00, F01, F03, G30, G31.0) | Race/ethnicity, smoking status, education level, body-mass index, physical activity level, number of children, and alcohol intake: questionnaire (survey 1)  Diabetes mellitus: questionnaire (survey 1-9), Medicare Benefits Schedule data (up to December 2020), Pharmaceutical Benefits Scheme data (up to August 2021), hospital admission data (up to December 2019), death registry data (up to December 2019)  Hypertension: questionnaire (survey 1-9)  Stroke: questionnaire (survey 1-9), hospital admission data (up to December 2019), death registry data (up to December 2019)  depression status: questionnaire (survey 2) |
| Prospect-EPIC  (Survey 1-5) | Questionnaire (survey 1): sub/infertility and infertility consult | Questionnaire (survey 1): number of miscarriages | Questionnaire (survey 1): number of stillbirths | Questionnaire (survey 4-5): diagnosis or treatment of dementia  Hospital admission data (up to December 2010): ICD 9 (290, 294.1, 294.2, 331.0, 331.1, 331.82) and ICD 10 (F00, F01, F02, F03, G30, G31.0, G31.83)  Death registry data (up to December 2010): ICD 9 (290.0, 290.1, 290.2, 290.4, 290.8, 290.9, 331.0, 331.1) and ICD 10 (F00, F01, F03, G30, G31.0) | Race/ethnicity, smoking status, education level, body-mass index, physical activity level, number of children, and alcohol intake: questionnaire (survey 1)  Diabetes mellitus: questionnaire (survey 1,4,5), hospital admission data (up to December 2010), death registry data (up to December 2010)  Hypertension: questionnaire (survey 1,4,5), death registry data (up to December 2010)  Stroke: questionnaire (survey 1-5), hospital admission data (up to December 2010), death registry data (up to December 2010) |
| UK Biobank  (Survey 1-4) | Not available | Questionnaire (survey 1-4): number of miscarriages | Questionnaire (survey 1-4): number of stillbirths | Assessment at center visit (visit 1-4): 1263 (dementia/Alzheimer/cognitive impairment)  Hospital admission data (up to January 2021): ICD 9 (290.0, 290.1, 290.2, 290.4, 290.8, 290.9, 331.0, 331.1) and ICD 10 (F00, F01, F03, G30, G31.0)  Death registry data (up to November 2021): ICD 9 (290.0, 290.1, 290.2, 290.4, 290.8, 290.9, 331.0, 331.1) and ICD 10 (F00, F01, F03, G30, G31.0) | Race/ethnicity, smoking status, education level, body-mass index, physical activity level, number of children, alcohol intake, and depression status: questionnaire (survey 1)  Diabetes mellitus, hypertension, and stroke: questionnaire (survey 1-4), assessment at center visit (visit 1-4), hospital admission data (up to January 2021), death registry data (up to November 2021) |
| WLH  (Survey 1-2) | Questionnaire (survey 1-2):  1. have difficulty in becoming pregnant for 1 or more years;  2.treatment of infertility | Not available | Not available | Hospital admission data (until 2010): ICD 9 (290.0, 290.1, 290.2, 290.4, 290.8, 290.9, 331.0, 331.1) and ICD 10 (F00, F01, F03, G30, G31.0) | Race/ethnicity, smoking status, education level, body-mass index, physical activity level, number of children: questionnaire (survey 1)  Diabetes mellitus, hypertension, and stroke: questionnaire (survey 1-2), patient registry data (up to 2012), and hospital admission data (until 2010)  Alcohol intake: questionnaire (survey 2)  Depression status: hospital admission data (until 2010) |

ALSWH-mid: Australian Longitudinal Study on Women’s Health; Prospect-EPIC: the Utrecht contribution to the European Prospective Investigation into Cancer and Nutrition cohort, the Netherlands; WLH: the Swedish Women’s Lifestyle and Health Study.

## **Table 3. Characteristics of women with and without dementia**

|  | Dementia | |
| --- | --- | --- |
| Characteristics | Never (N=287,721) | Ever (N=3,334) |
| History of infertility, No. (%) |  |  |
| No | 54,597 (82.1%) | 431 (86.0%) |
| Yes | 11,912 (17.9%) | 70 (14.0%) |
| History of miscarriage, No. (%) |  |  |
| No | 180,011 (74.6%) | 2,423 (76.0%) |
| Yes | 61,410 (25.4%) | 764 (24.0%) |
| Number of miscarriages, No. (%) |  |  |
| 0 | 180,011 (74.6%) | 2,423 (76.1%) |
| 1 | 43,793 (18.1%) | 524 (16.5%) |
| 2 | 11,295 (4.7%) | 139 (4.4%) |
| ≥3 | 6,272 (2.6%) | 97 (3.1%) |
| History of stillbirth, No. (%) |  |  |
| No | 224,508 (96.8%) | 2,851 (95.2%) |
| Yes | 7,443 (3.2%) | 145 (4.8%) |
| Number of stillbirths, No. (%) |  |  |
| 0 | 224,508 (96.8%) | 2,851 (95.2%) |
| 1 | 6,463 (2.8%) | 117 (3.9%) |
| ≥2 | 970 (0.4%) | 28 (0.9%) |
| Race/ethnicity, No. (%) |  |  |
| Caucasian | 276,082 (96.0%) | 3,220 (96.6%) |
| Others | 11,639 (4.1%) | 114 (3.4%) |
| Education level (years), No. (%) |  |  |
| ≤10 | 138,998 (48.3%) | 2,160 (64.8%) |
| 11-12 | 46,660 (16.2%) | 399 (12.0%) |
| >12 | 102,063 (35.5%) | 775 (23.3%) |
| Smoking status, No. (%) |  |  |
| Never or past smoker | 253,530 (88.1%) | 2,967 (89.0%) |
| Current smoker | 34,191 (11.9%) | 367 (11.0%) |
| Body-mass index, No. (%) |  |  |
| Underweight | 2,491 (0.9%) | 32 (1.0%) |
| Normal | 124,254 (43.2%) | 1,193 (35.8%) |
| Overweight | 100,700 (35.0%) | 1,271 (38.1%) |
| Obese | 60,276 (21.0%) | 838 (25.1%) |
| Hypertension, No. (%) |  |  |
| No | 188,013 (65.4%) | 1,280 (38.4%) |
| Yes | 99,708 (34.7%) | 2,054 (61.6%) |
| Diabetes mellitus, No. (%) |  |  |
| No | 265,365 (92.2%) | 2,654 (79.6%) |
| Yes | 22,356 (7.8%) | 680 (20.4%) |
| Stroke, No. (%) |  |  |
| No | 278,771 (96.9%) | 2,849 (85.5%) |
| Yes | 8,950 (3.1%) | 485 (14.6%) |

## **Table 4. Characteristics of women included and excluded from the analyses**

|  | Infertility and dementia | | Miscarriage and dementia | | Stillbirth and dementia | |
| --- | --- | --- | --- | --- | --- | --- |
|  | Included | Excluded | Included | Excluded | Included | Excluded |
| Sample size, No. (%) | 67,010 (83.4) | 13,321 (16.6) | 244,608 (94.2) | 14,950 (5.8) | 234,947 (95.3) | 11,673 (4.7) |
| Race/ethnicity, No. (%) |  |  |  |  |  |  |
| Caucasian | 66,665 (99.5) | 13,010 (97.7) | 232,996 (95.3) | 13,573 (90.8) | 223,754 (95.2) | 10,466 (89.7) |
| Others | 345 (0.5) | 311 (2.3) | 11,612 (4.8) | 1,377 (9.2) | 11,193 (4.8) | 1,207 (10.3) |
| Education level, No. (%) |  |  |  |  |  |  |
| ≤10 years | 23,851 (35.6) | 5,378 (42.9) | 127,133 (52.0) | 7,639 (55.1) | 122,247 (52.0) | 6,004 (56.1) |
| 11-12 years | 19,311 (28.8) | 3,281 (26.2) | 34,012 (13.9) | 2,185 (15.8) | 32,403 (13.8) | 1,637 (15.3) |
| >12 years | 23,848 (35.6) | 3,877 (30.9) | 83,463 (34.1) | 4,032 (29.1) | 80,297 (34.2) | 3,065 (28.6) |
| Smoking status, No. (%) |  |  |  |  |  |  |
| Never or past smoker | 53,046 (79.2) | 9,906 (77.7) | 219,956 (89.9) | 11,607 (85.3) | 212,059 (90.3) | 9,309 (86.5) |
| Current smoker | 13,964 (20.8) | 2,844 (22.3) | 24,652 (10.1) | 2,000 (14.7) | 22,888 (9.7) | 1,449 (13.5) |
| Body-mass index, No. (%) |  |  |  |  |  |  |
| Underweight | 990 (1.5) | 194 (1.9) | 1,725 (0.7) | 86 (0.9) | 1,551 (0.7) | 46 (0.7) |
| Normal | 41,379 (61.8) | 5,916 (57.7) | 93,585 (38.3) | 3,563 (37.4) | 88,817 (37.8) | 2,292 (32.9) |
| Overweight | 18,093 (27.0) | 2,828 (27.6) | 91,375 (37.4) | 3,518 (37.0) | 88,523 (37.7) | 2,745 (39.5) |
| Obese | 6,548 (9.8) | 1,312 (12.8) | 57,923 (23.7) | 2,350 (24.7) | 56,056 (23.9) | 1,876 (27.0) |
| Hypertension |  |  |  |  |  |  |
| No | 45,105 (67.3) | 8,810 (66.2) | 153,882 (62.9) | 8,295 (55.5) | 148,952 (63.4) | 6,578 (56.4) |
| Yes | 21,905 (32.7) | 4,507 (33.8) | 90,726 (37.1) | 6,655 (44.5) | 85,995 (36.6) | 5,095 (43.7) |
| Diabetes mellitus |  |  |  |  |  |  |
| No | 60,514 (90.3) | 11,594 (87.0) | 225,178 (92.1) | 13,018 (87.1) | 217,478 (92.6) | 10,440 (89.4) |
| Yes | 6,496 (9.7) | 1,726 (13.0) | 19,430 (7.9) | 1,932 (12.9) | 17,469 (7.4) | 1,233 (10.6) |
| Stroke |  |  |  |  |  |  |
| No | 64,528 (96.3) | 12,536 (94.1) | 236,348 (96.6) | 13,739 (91.9) | 227,237 (96.7) | 10,681 (91.5) |
| Yes | 2,482 (3.7) | 784 (5.9) | 8,260 (3.4) | 1,211 (8.1) | 7,710 (3.3) | 992 (8.5) |

Distributions between complete dataset and the dataset with missing value were compared using Chi-square test. All the p value were <.0001, except the p values for smoking status and hypertension between women included and excluded in the analysis for infertility and dementia (p=0.0002 for smoking status and p=0.0096 for hypertension), the p value for body-mass index between women included and excluded in the analysis for miscarriage and dementia (p=0.0117 for body-mass index).

# **Kaplan-Meier survival plot with log-rank test**

## **Figure 2. Kaplan-Meier survival curves comparing the rate of dementia according to the history of infertility**


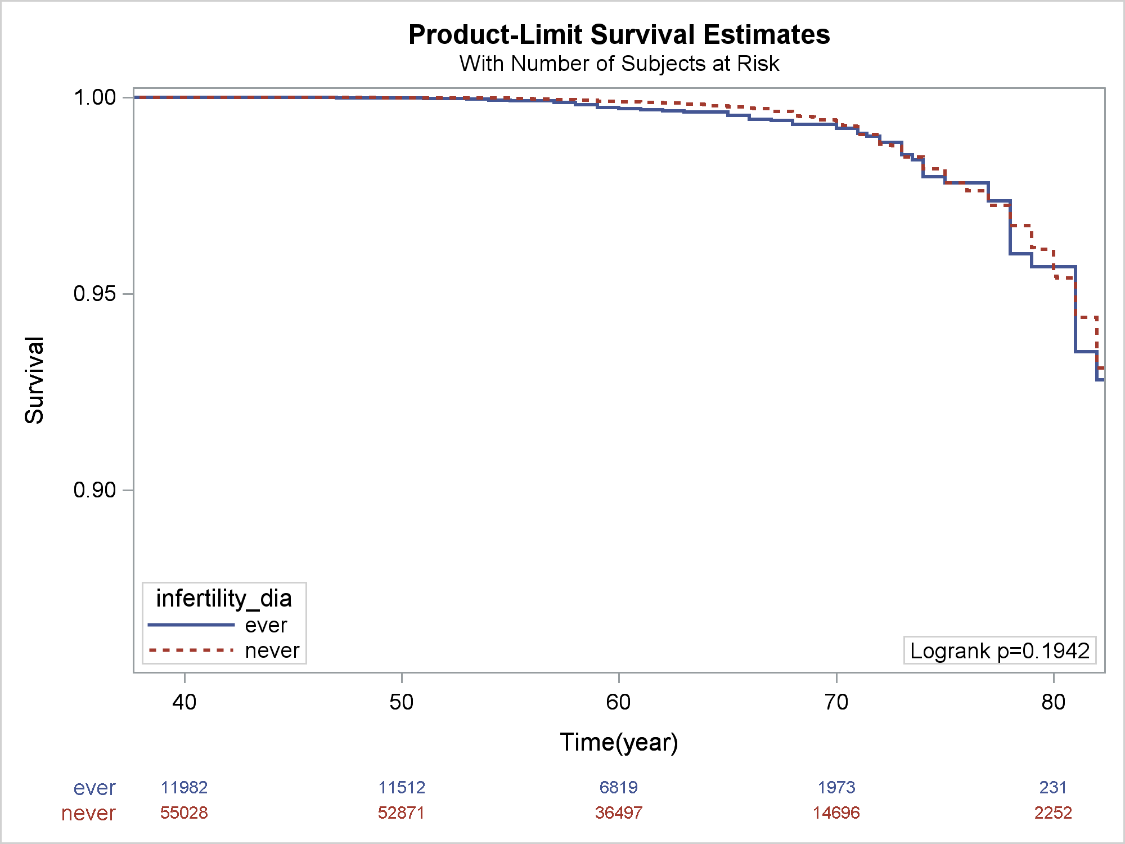


## **Figure 3. Kaplan-Meier survival curves comparing the rate of dementia according to the history of miscarriage**


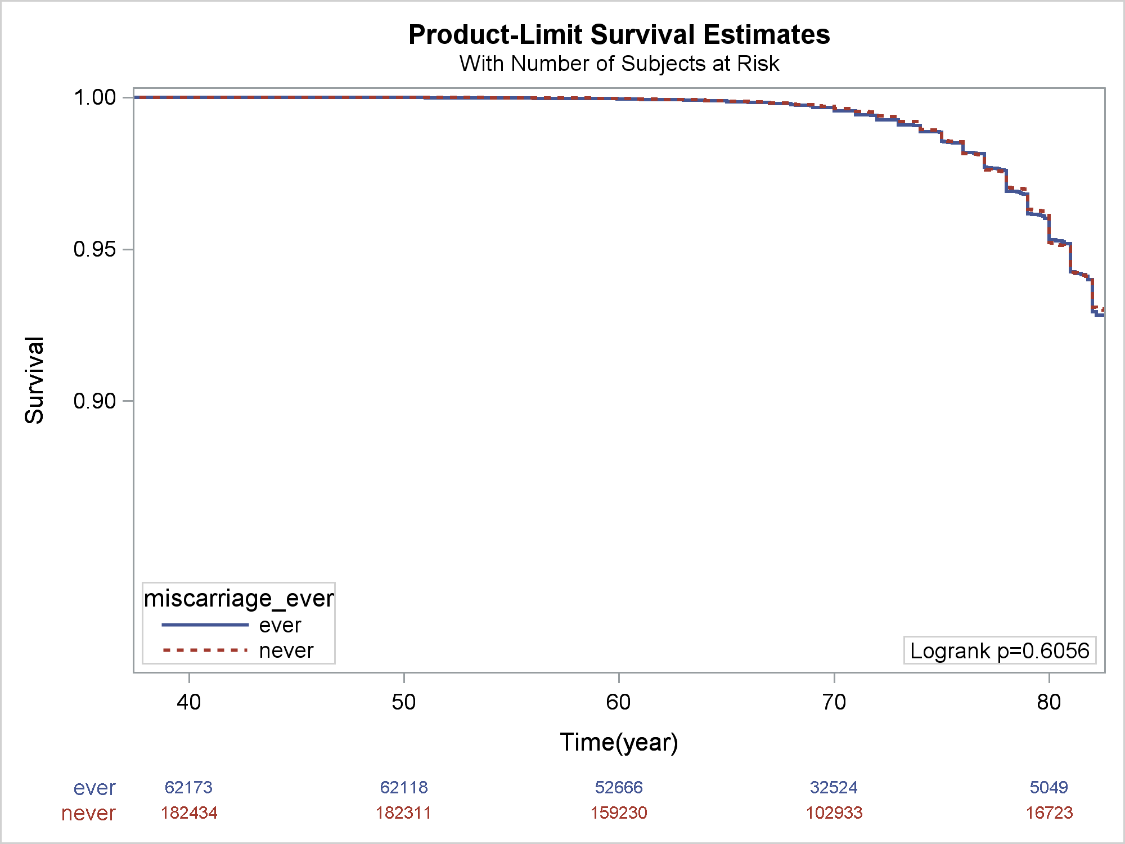


## **Figure 4. Kaplan-Meier survival curves comparing the rate of dementia according to the number of miscarriages**


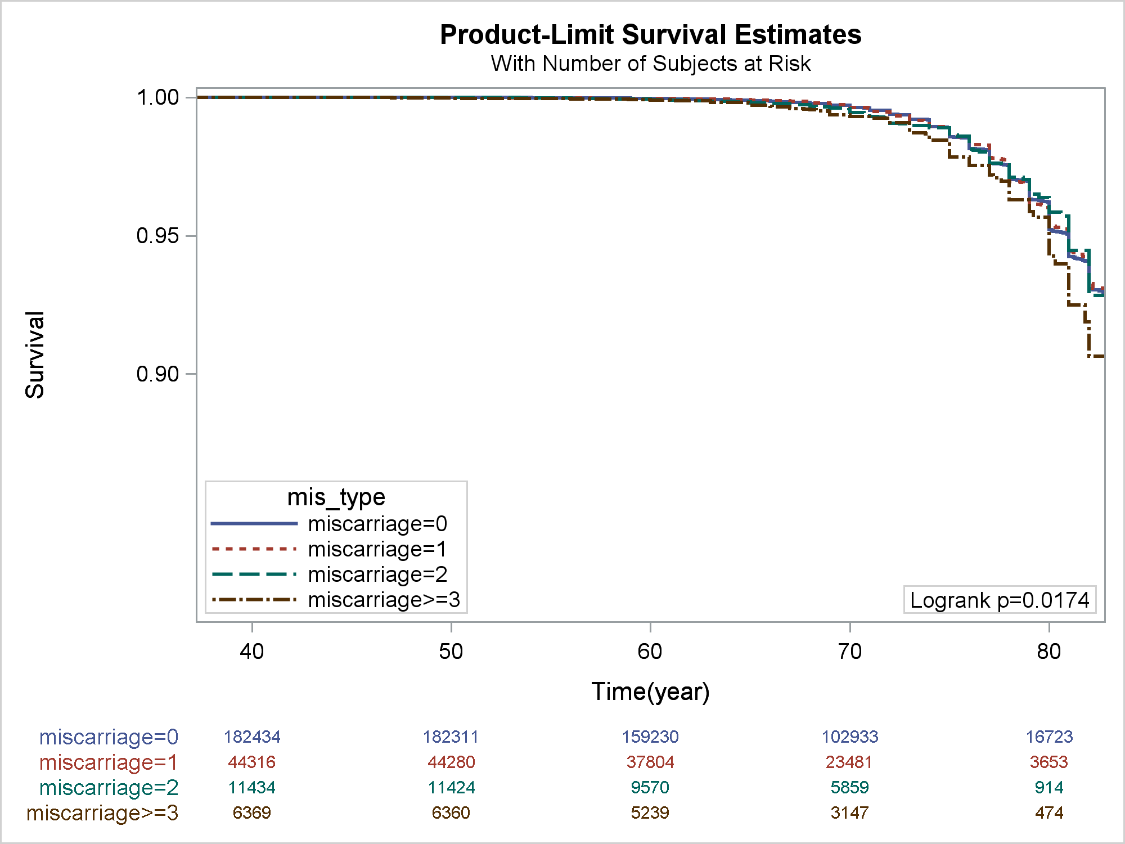


## **Figure 5. Kaplan-Meier survival curves comparing the rate of dementia according to the history of stillbirth**


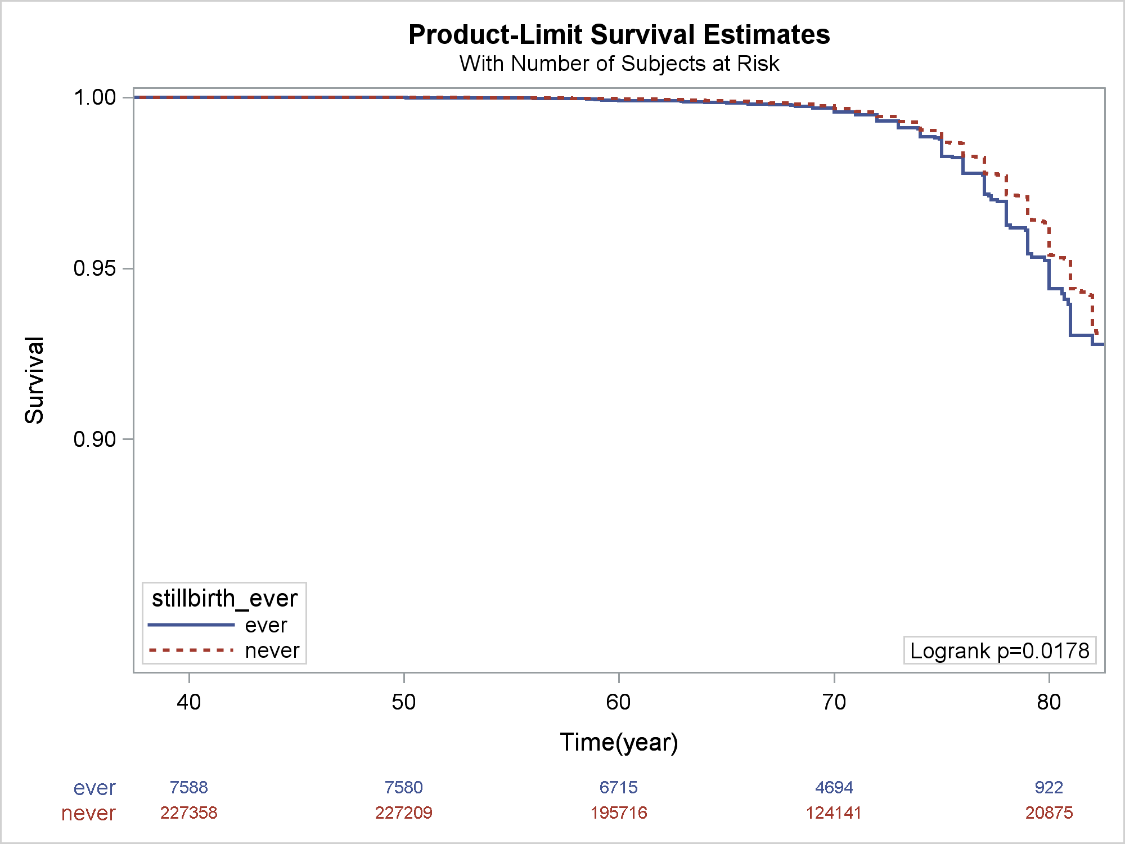


## **Figure 6. Kaplan-Meier survival curves comparing the rate of dementia according to the number of stillbirths**


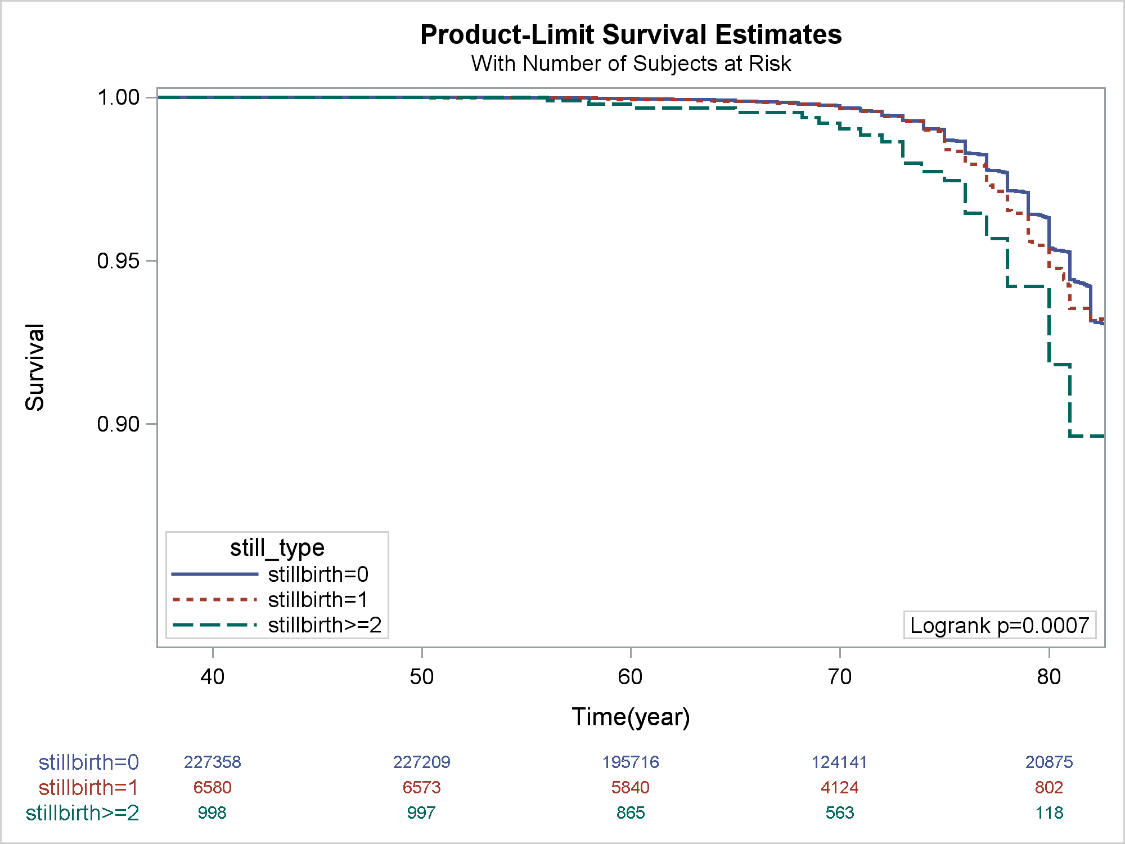


# **Sensitivity analyses**

## **Table 5. Sensitivity analysis for the association between infertility, miscarriage, stillbirth, and dementia estimated by Fine and Gray competing risk model**

| Exposure |  | Person-year | Sample size | Event | IR | Crude model  HR (95%CI) | Adjusted model  HR (95%CI) |
| --- | --- | --- | --- | --- | --- | --- | --- |
| Infertility | Never | 3,504,824 | 55,028 | 431 | 1.23 | Ref. | Ref. |
|  | Ever | 737,923 | 11,982 | 70 | 0.95 | 1.06 (0.76,1.48) | 1.06 (0.77,1.45) |
| Miscarriage | Never | 12,738,716 | 182,434 | 2,423 | 1.90 | Ref. | Ref. |
|  | Ever | 4,291,693 | 62,174 | 764 | 1.78 | 0.98 (0.95,1.01) | 0.97 (0.94,1.00) |
|  | 0 | 12,738,716 | 182,434 | 2,423 | 1.90 | Ref. | Ref. |
|  | 1 | 3,065,128 | 44,317 | 524 | 1.71 | 0.94 (0.91,0.97) | 0.94 (0.92,0.96) |
|  | 2 | 786,986 | 11,434 | 139 | 1.77 | 0.96 (0.83,1.10) | 0.94 (0.82,1.09) |
|  | ≥3 | 435,644 | 6,369 | 97 | 2.23 | 1.26 (1.18,1.34) | 1.18 (1.14,1.22) |
| Stillbirth | Never | 15,815,662 | 227,359 | 2,851 | 1.80 | Ref. | Ref. |
|  | Ever | 537,885 | 7,588 | 145 | 2.70 | 1.20 (1.11,1.30) | 1.08 (1.02,1.14) |
|  | 0 | 15,815,662 | 227,359 | 2,851 | 1.80 | Ref. | Ref. |
|  | 1 | 467,222 | 6,580 | 117 | 2.50 | 1.11 (1.04,1.19) | 1.01 (0.97,1.05) |
|  | ≥2 | 69,911 | 998 | 28 | 4.01 | 1.85 (1.73,1.98) | 1.57 (1.42,1.73) |

IR: incidence rate (per 10,000 person-year). HR: hazard ratio. CI: confidence interval. Models were adjusted for race, education level, smoking status, body-mass index, hypertension, diabetes, and stroke, when hypertension, diabetes, and stroke were included as time-varying covariates. Study variability and within-study correlation were taken into account by including study as a covariate and using robust variance estimators in all models.

## **Table 6. Sensitivity analysis for the association between infertility and dementia restricting to women with children**

| Exposure |  | Person-year | Sample size | Event | IR | Crude model  HR (95%CI) | Adjusted model  HR (95%CI) |
| --- | --- | --- | --- | --- | --- | --- | --- |
| Infertility | Never | 3,210,993 | 50,472 | 393 | 1.22 | Ref. | Ref. |
|  | Ever | 591,973 | 9,661 | 58 | 0.98 | 1.16 (0.79,1.71) | 1.17 (0.82,1.67) |
| Miscarriage | Never | 12,346,105 | 176,419 | 2,393 | 1.94 | Ref. | Ref. |
|  | Ever | 4,012,878 | 57,964 | 728 | 1.81 | 0.98 (0.95,1.01) | 0.97 (0.95,0.99) |
|  | 0 | 12,346,105 | 176,419 | 2,393 | 1.94 | Ref. | Ref. |
|  | 1 | 2,875,099 | 41,445 | 502 | 1.75 | 0.94 (0.90,0.97) | 0.94 (0.91,0.97) |
|  | 2 | 737,959 | 10,695 | 133 | 1.80 | 0.98 (0.86,1.11) | 0.96 (0.83,1.10) |
|  | ≥3 | 396,326 | 5,776 | 89 | 2.25 | 1.27 (1.19,1.36) | 1.19 (1.14,1.23) |
| Stillbirth | Never | 15,193,389 | 217,846 | 2,798 | 1.84 | Ref. | Ref. |
|  | Ever | 514,397 | 7,243 | 136 | 2.64 | 1.17 (1.09,1.27) | 1.05 (1.00,1.11) |
|  | 0 | 15,193,389 | 217,846 | 2,798 | 1.84 | Ref. | Ref. |
|  | 1 | 449,443 | 6,319 | 110 | 2.45 | 1.08 (1.01,1.15) | 0.97 (0.94,1.01) |
|  | ≥2 | 64,201 | 914 | 26 | 4.05 | 1.89 (1.76,2.01) | 1.61 (1.43,1.82) |

IR: incidence rate (per 10,000 person-year). HR: hazard ratio. CI: confidence interval. Models were adjusted for race, education level, smoking status, body-mass index, hypertension, diabetes, and stroke, when hypertension, diabetes, and stroke were included as time-varying covariates. Study variability and within-study correlation were taken into account by including study as a covariate and using robust variance estimators in all models. Compared to main analysis, 6877, 10,225, and 9858 women, who did not have children or had missing value on the number of children, were excluded from the analysis of infertility, miscarriage, and stillbirth respectively.

## **Table 7. Sensitivity analysis for the association between infertility and dementia restricting to women without gestational hypertension or gestational diabetes**

| Exposure |  | Person-year | Sample size | Event | IR | Crude model  HR (95%CI) | Adjusted model  HR (95%CI) |
| --- | --- | --- | --- | --- | --- | --- | --- |
| Infertility | Never | 2,592,079 | 42,044 | 217 | 0.84 | Ref. | Ref. |
|  | Ever | 593,364 | 9,880 | 45 | 0.76 | 1.28 (0.90,1.84) | 1.28 (0.94,1.74) |
| Miscarriage | Never | 11,895,126 | 170,340 | 2,227 | 1.87 | Ref. | Ref. |
|  | Ever | 3,862,638 | 55,984 | 662 | 1.71 | 0.98 (0.94,1.03) | 0.97 (0.93,1.01) |
|  | 0 | 11,895,126 | 170,340 | 2,227 | 1.87 | Ref. | Ref. |
|  | 1 | 2,771,850 | 40,091 | 453 | 1.63 | 0.93 (0.88,0.97) | 0.93 (0.88,0.97) |
|  | 2 | 703,987 | 10,238 | 123 | 1.75 | 1.01 (0.91,1.12) | 1.00 (0.90,1.10) |
|  | ≥3 | 383,984 | 5,616 | 82 | 2.14 | 1.30 (1.22,1.37) | 1.22 (1.19,1.24) |
| Stillbirth | Never | 15,255,831 | 219,248 | 2,752 | 1.80 | Ref. | Ref. |
|  | Ever | 510,674 | 7,199 | 142 | 2.78 | 1.26 (1.21,1.30) | 1.13 (1.11,1.14) |
|  | 0 | 15,255,831 | 219,248 | 2,752 | 1.80 | Ref. | Ref. |
|  | 1 | 443,847 | 6,246 | 115 | 2.59 | 1.16 (1.15,1.17) | 1.05 (1.03,1.06) |
|  | ≥2 | 66,226 | 945 | 27 | 4.08 | 1.96 (1.79,2.14) | 1.73 (1.58,1.89) |

IR: incidence rate (per 10,000 person-year). HR: hazard ratio. CI: confidence interval. Models were adjusted for race, education level, smoking status, body-mass index, hypertension, diabetes, and stroke, when hypertension, diabetes, and stroke were included as time-varying covariates. Study variability and within-study correlation were taken into account by including study as a covariate and using robust variance estimators in all models. Compared to main analysis, 15,086, 18,284, and 8500 women, who had gestational hypertension or gestational diabetes, or had missing value on these two pregnancy complications, were excluded from the analysis of infertility, miscarriage, and stillbirth, respectively.

## **Table 8. Sensitivity analysis for the association between miscarriage and dementia with additional adjustment of age at last birth, alcohol intake and depression status**

| Exposure |  | Person-year | Sample size | Event | IR | Model 1  HR (95%CI) | Model 2  HR (95%CI) | Model 3  HR (95%CI) | Model 4  HR (95%CI) | Model 5  HR (95%) |
| --- | --- | --- | --- | --- | --- | --- | --- | --- | --- | --- |
| Infertility | Never | 1,439,604 | 23,526 | 142 | 0.99 | Ref. | Ref. | Ref. | Ref. | Ref. |
|  | Ever | 304,948 | 5,094 | 33 | 1.08 | 1.60 (0.79,3.22) | 1.56 (0.78,3.12) | 1.54 (0.74,3.19) | 1.54 (0.74,3.19) | 1.51 (0.72,3.17) |
| Miscarriage | Never | 11,180,059 | 160,164 | 2,075 | 1.86 | Ref. | Ref. | Ref. | Ref. | Ref. |
|  | Ever | 3,615,372 | 52,443 | 618 | 1.71 | 0.98 (0.98,0.99) | 0.97 (0.97,0.97) | 0.97 (0.96,0.97) | 0.97 (0.96,0.97) | 0.96 (0.96,0.97) |
|  | 0 | 11,180,059 | 160,164 | 2,075 | 1.86 | Ref. | Ref. | Ref. | Ref. | Ref. |
|  | 1 | 2,592,957 | 37,523 | 431 | 1.66 | 0.94 (0.90,0.98) | 0.93 (0.90,0.97) | 0.93 (0.90,0.96) | 0.94 (0.91,0.97) | 0.93 (0.90,0.96) |
|  | 2 | 663,454 | 9,666 | 114 | 1.72 | 1.02 (0.87,1.19) | 1.01 (0.85,1.20) | 1.01 (0.84,1.20) | 1.00 (0.84,1.20) | 1.00 (0.83,1.19) |
|  | ≥3 | 358,960 | 5,254 | 73 | 2.03 | 1.25 (1.25,1.26) | 1.16 (1.13,1.19) | 1.15 (1.13,1.17) | 1.14 (1.11,1.17) | 1.13 (1.10,1.15) |
| Stillbirth | Never | 13,783,429 | 198,245 | 2,428 | 1.76 | Ref. | Ref. | Ref. | Ref. | Ref. |
|  | Ever | 457,492 | 6,462 | 117 | 2.56 | 1.20 (1.00,1.44) | 1.08 (0.89,1.29) | 1.07 (0.89,1.29) | 1.06 (0.88,1.27) | 1.05 (0.87,1.27) |
|  | 0 | 13,783,429 | 198,245 | 2,428 | 1.76 | Ref. | Ref. | Ref. | Ref. | Ref. |
|  | 1 | 399,899 | 5,640 | 95 | 2.38 | 1.11 (0.90,1.36) | 1.00 (0.81,1.22) | 1.00 (0.81,1.22) | 0.98 (0.80,1.21) | 0.98 (0.80,1.20) |
|  | ≥2 | 57,593 | 822 | 22 | 3.82 | 1.89 (1.24,2.87) | 1.66 (1.09,2.53) | 1.65 (1.08,2.51) | 1.57 (1.03,2.39) | 1.57 (1.03,2.39) |

IR: incidence rate (per 10,000 person-year). HR: hazard ratio. CI: confidence interval. Model 1: model with exposure. Model 2: model was additionally adjusted for race, education level, smoking status, body-mass index, hypertension, diabetes, and stroke, when hypertension, diabetes, and stroke were included as time-varying covariates. Model 3: model was adjusted for age at last birth and factors in model 2. Model 4: model was adjusted for alcohol intake and factors in model 3. Model 5: model was adjusted for depression status and factors in model 4. Study variability and within-study correlation were taken into account by including study as a covariate and using robust variance estimators in all models. Compared to main analysis, 38,390, 32,001, and 30,240 women, who had missing value on age at last birth, alcohol intake or depression status, were excluded from the analysis of infertility, miscarriage, and stillbirth respectively.

# **Association in single study**

## **Figure 7. Association between infertility and dementia in each study**


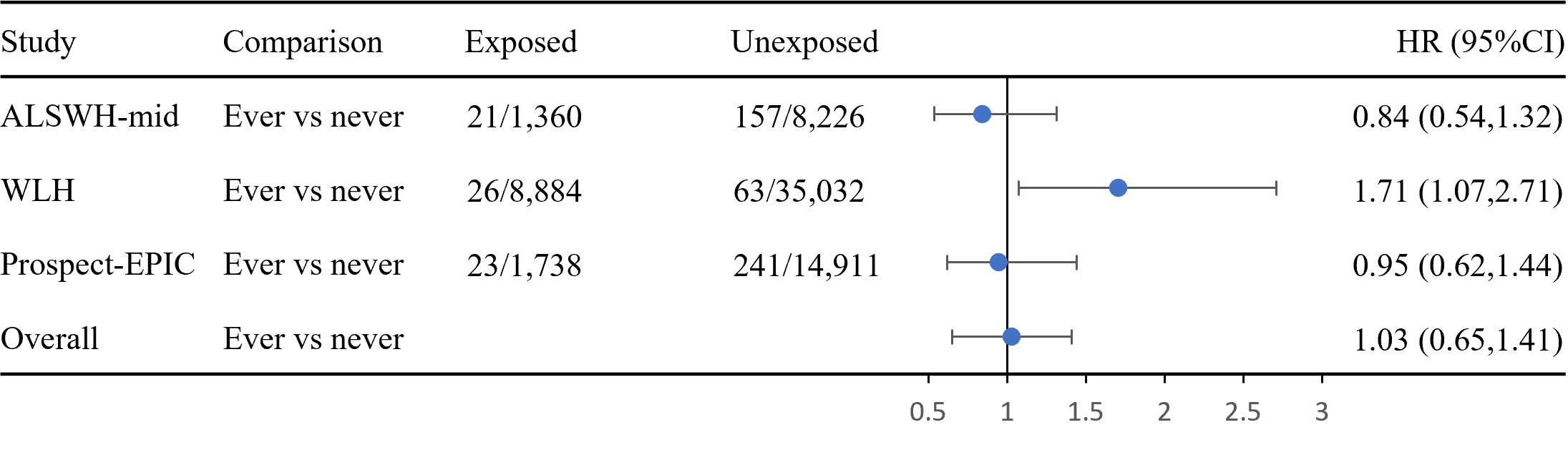


HRs in single study were adjusted for race, education level, smoking status, body-mass index, hypertension, diabetes mellitus, and stroke, when hypertension, diabetes, and stroke were included as time-varying covariates. Heterogeneity: I^2^=43.5%, p=0.170 for infertility (ever vs never).

## **Figure 8. Association between miscarriage and dementia in each study**


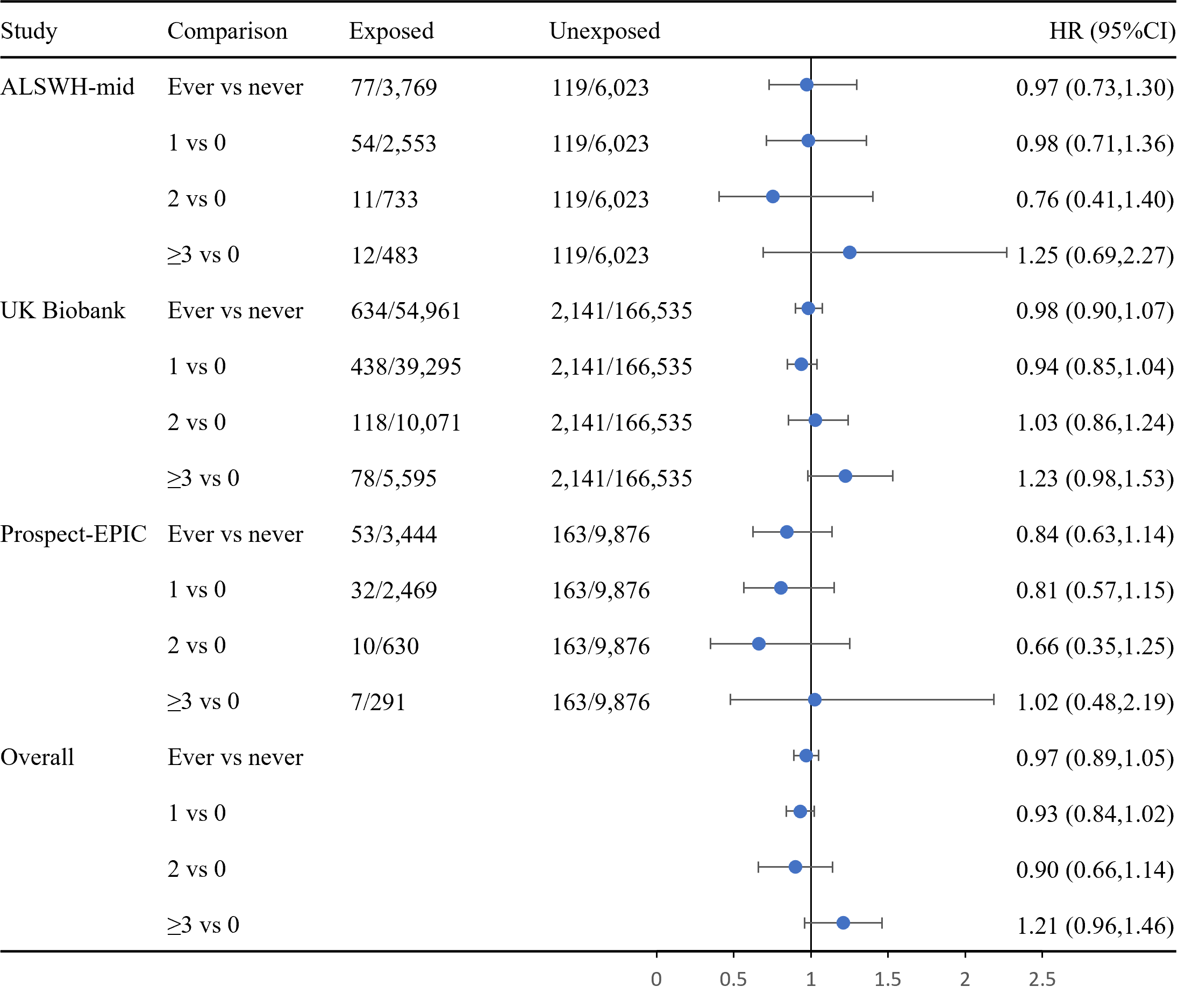


HRs in single study were adjusted for race, education level, smoking status, body-mass index, hypertension, diabetes mellitus, and stroke, when hypertension, diabetes, and stroke were included as time-varying covariates. Heterogeneity: I^2^=0.0%, p=0.601 for miscarriage (ever vs never); I^2^=0.0%, p=0.668 for miscarriage (1 vs 0); I^2^=29.1%, P=0.244 for miscarriage (2 vs 0); I^2^=0.0%, p=0.903 for miscarriage (≥3 vs 0).

## **Figure 9. Association between stillbirth and dementia in each study**


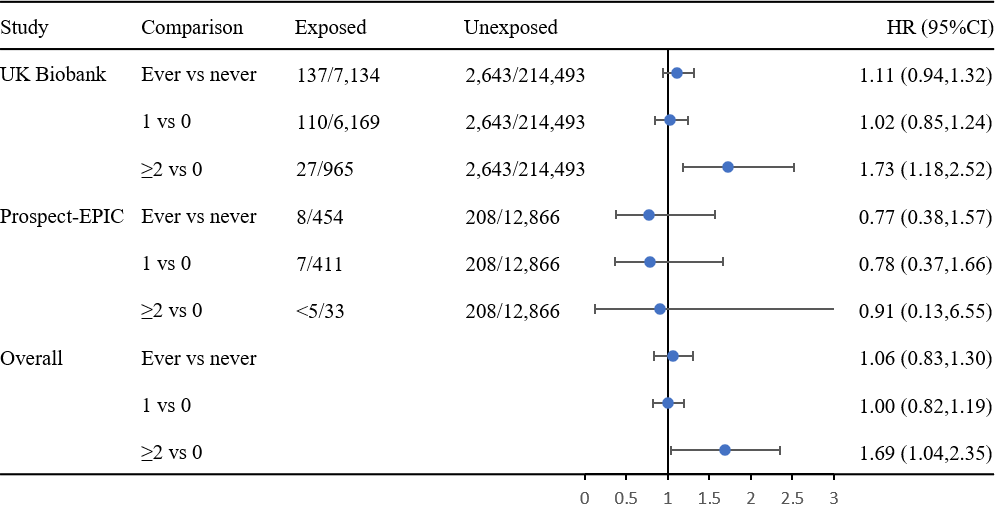


HRs in single study were adjusted for race, education level, smoking status, body-mass index, hypertension, diabetes mellitus, and stroke, when hypertension, diabetes, and stroke were included as time-varying covariates. Heterogeneity: I^2^=11.9%, p=0.287 for stillbirth (ever vs never); I^2^=0.0%, p=0.483 for stillbirth (1 vs 0); I^2^=0.0%, p=0.628 for stillbirth (≥2 vs 0).

**Reference**

1. Hao W, Fu C, Dong C, et al. Age at menopause and all-cause and cause-specific dementia: a prospective analysis of the UK Biobank cohort. *Hum Reprod*. 2023;38(9):1746-1754. doi:10.1093/humrep/dead130

2. Fu C, Hao W, Ma Y, et al. Number of live births, age at the time of having a child, span of births and risk of dementia: a population-based cohort study of 253,611 U.K. women. *J Women’s Heal*. 2023;32(6):680-692. doi:10.1089/jwh.2022.0396

3. DiBiase RM, Gottesman RF, Tom SE, et al. Parity and risk of dementia in women: the atherosclerosis risk in communities study. *J Women’s Heal*. 2023;32(10):1031-1040. doi:10.1089/jwh.2023.0030

4. Gemmill A, Weiss J. The relationship between fertility history and incident dementia in the U.S. health and retirement study. Carr D, ed. *Journals Gerontol Ser B*. 2022;77(6):1118-1131. doi:10.1093/geronb/gbab183

5. Gong J, Harris K, Peters SAE, Woodward M. Reproductive factors and the risk of incident dementia: a cohort study of UK Biobank participants. Brayne C, ed. *PLOS Med*. 2022;19(4):e1003955. doi:10.1371/journal.pmed.1003955

6. Andolf E, Bladh M, Möller L, Sydsjö G. Prior placental bed disorders and later dementia: a retrospective Swedish register‐based cohort study. *BJOG An Int J Obstet Gynaecol*. 2020;127(9):1090-1099. doi:10.1111/1471-0528.16201

7. Yoo JE, Shin DW, Han K, et al. Female reproductive factors and the risk of dementia: a nationwide cohort study. *Eur J Neurol*. 2020;27(8):1448-1458. doi:10.1111/ene.14315

8. Bae JB, Lipnicki DM, Han JW, et al. Parity and the risk of incident dementia: a COSMIC study. *Epidemiol Psychiatr Sci*. 2020;29:e176. doi:10.1017/S2045796020000876

9. Bae J Bin, Lipnicki DM, Han JW, et al. Does parity matter in women’s risk of dementia? A COSMIC collaboration cohort study. *BMC Med*. 2020;18(1):210. doi:10.1186/s12916-020-01671-1

10. Basit S, Wohlfahrt J, Boyd HA. Pregnancy loss and risk of later dementia: a nationwide cohort study, Denmark, 1977–2017. *Alzheimer’s Dement Transl Res Clin Interv*. 2019;5(1):146-153. doi:10.1016/j.trci.2019.02.006

11. Gilsanz P, Lee C, Corrada MM, Kawas CH, Quesenberry CP, Whitmer RA. Reproductive period and risk of dementia in a diverse cohort of health care members. *Neurology*. 2019;92(17). doi:10.1212/WNL.0000000000007326

12. Prince MJ, Acosta D, Guerra M, et al. Reproductive period, endogenous estrogen exposure and dementia incidence among women in Latin America and China; A 10/66 population-based cohort study. El Bcheraoui C, ed. *PLoS One*. 2018;13(2):e0192889. doi:10.1371/journal.pone.0192889

13. Geerlings MI. Reproductive period and risk of dementia in postmenopausal women. *JAMA*. 2001;285(11):1475. doi:10.1001/jama.285.11.1475

14. Jang H, Bae JB, Dardiotis E, et al. Differential effects of completed and incomplete pregnancies on the risk of Alzheimer disease. *Neurology*. 2018;91(7):e643-e651. doi:10.1212/WNL.0000000000006000
